# Distinct Roles of ComK1 and ComK2 in Gene Regulation in *Bacillus cereus*

**DOI:** 10.1371/journal.pone.0021859

**Published:** 2011-07-01

**Authors:** Aleksandra M. Mirończuk, Amagoia Maňu, Oscar P. Kuipers, Ákos T. Kovács

**Affiliations:** 1 Molecular Genetics Group, Groningen Biomolecular Sciences and Biotechnology Institute, University of Groningen, Groningen, The Netherlands; 2 Kluyver Centre for Genomics of Industrial Fermentation, Groningen, The Netherlands; Tulane University, United States of America

## Abstract

The *B. subtilis* transcriptional factor ComK regulates a set of genes coding for DNA uptake from the environment and for its integration into the genome. In previous work we showed that *Bacillus cereus* expressing the *B. subtilis* ComK protein is able to take up DNA and integrate it into its own genome. To extend our knowledge on the effect of *B. subtilis* ComK overexpression in *B. cereus* we first determined which genes are significantly altered. Transcriptome analysis showed that only part of the competence gene cluster is significantly upregulated. Two ComK homologues can be identified in *B. cereus* that differ in their respective homologies to other ComK proteins. ComK1 is most similar, while ComK2 lacks the C-terminal region previously shown to be important for transcription activation by *B. subtilis* ComK. *comK1* and *comK2* overexpression and deletion studies using transcriptomics techniques showed that ComK1 enhances and ComK2 decreases expression of the *comG* operon, when *B. subtilis* ComK was overexpressed simultaneously.

## Introduction

*Bacillus cereus* is a foodborne pathogenic bacterium and a common contaminant of food and dairy products. This gram-positive, spore-forming bacterium is an agent of two types of foodborne diseases, the emetic and the diarrheal forms. The most important virulence factors are heat-stable emetic toxins and enterotoxins. Symptoms are commonly mild and self-limiting, from diarrhea to vomiting [1]. *B. cereus* can also cause severe infections, especially in immumocompromised patients [2]. To survive in changing environments and under stress conditions, bacteria evolved adaptive networks related to e.g. biofilm formation, spore formation or competence development. Competence is defined as a physiological state of bacteria in which exogenous DNA can be incorporated leading to a genetic transformation event. Whole genome sequences of the *Bacillus* group showed that the presence of competence genes is not restricted to *B. subtilis* and closely related species, but is apparent throughout the *Bacillus* genus. Homologues of most structural proteins required for transformation in *B. subtilis* have been found in *B. cereus*, with the exception of clear homologues for the ComGE, ComGF, ComGG proteins [3], [4], although the presence of functional homologues has been suggested [5]. Interestingly, under laboratory conditions only a fraction of cells become competent, ranging between 10–20% of the population in the model organism *B. subtilis* [6]. Competence for genetic transformation in *B. subtilis* is elaborately regulated, and pivotal to this process is the level of the ComK protein. This protein is an activator of the so called ComK-regulon that also comprises all late-competence genes that are required for transformation [7], [8]. ComK activity is controlled by multiple mechanisms, such as quorum sensing, proteolytic degradation by the MecA/ClpCP complex, and transcriptional control by multiple transcription factors [9]–[11]. Premature transcription of *comK* is prevented by three different repressors: AbrB, CodY and Rok, which all bind to the *comK* promoter region. In addition, during the exponential growth phase, the small amount of ComK that is produced, is trapped by MecA, which targets it for proteolytic degradation by the ClpCP proteasome complex [11], [12]. When the competence quorum sensing mechanism is activated at the end of exponential growth, the small protein ComS is produced and liberates ComK from the MecA complex [9], [13]. Subsequently, ComK activates a number of promoters including its own [14], [15]. Among the known ComK targets are genes involved in the DNA uptake machinery [16].

In *B. cereus* ATCC 14579 two homologues of ComK (BC1134, hereafter referred to as ComK1; BC5250 as ComK2) could be identified [5]. Notably, *in silico* analysis demonstrated that these two putative ComK proteins have a different level of homology to *B. subtilis* ComK, i.e. ComK1 61%, whereas ComK2 shows only 44%. The ComK1 protein is similar in length to the *B. subtilis* ComK protein, while the ComK2 protein of *B. cereus* appears to be C-terminally truncated by 22 aminoacids. Taken together, this data suggests that the regulation of gene expression by ComK proteins in *B. cereus* differs significantly from that known in *B. subtilis*.

In a recent study we reported that a minimal system for functional DNA uptake exists in *B. cereus* [17]. We introduced the *B. subtilis comK* gene (hereafter referred to as *comK_Bsu_*) under the IPTG-inducible hyper-spank promoter (pNWcomK_Bsu_) into the *B. cereus* ATCC 14579 strain. After induction of *comK_Bsu_*, cells grown in minimal medium displayed modest functional DNA uptake, as genomic DNA or plasmid DNA was shown to be taken up by the cells. This DNA uptake is less efficient compared to *B. subtilis* and *B. licheniformis*, where overexpression of their own *comK*s resulted in highly efficient genetic competence [18], [19].

Here, we investigate the effect of the overexpression of various *comK* genes in *B. cereus*. Our study reveals that upon overexpression of *comK_Bsu_* in *B. cereus* only part of the late competence gene cluster is upregulated. Further, we explore the roles of ComK1 and ComK2 in *B. cereus*. While we observe no genes to be differentially expressed connected to competence when either *comK1* or *comK2* are overexpressed, interestingly, ComK1 and ComK2 influence *comGA* expression under *comK_Bsu_* inducing conditions.

## Results

### Transcriptome changes in *B. cereus* by overexpression of *comK_Bsu_*

We previously showed that *B. cereus* contains a minimal functional DNA uptake apparatus and is able to integrate exogenous DNA into its own chromosome [17]. Under these conditions the late competence gene *comGA* was shown to be induced upon *comK_Bsu_* overexpression. We investigated the impact of comK*_Bsu_* overexpression using transcriptomics. DNA microarray analysis was used to compare the transcriptional profiles of *B. cereus* ATCC 14579 containing plasmid pNW33N (empty vector) with those of *B. cereus* ATCC 14579 containing plasmid pNWcomK_Bsu_ (*comK_Bsu_* overexpression) grown in MM medium. Three independent cultures were used for both the control and the target strains in this experiment. Samples were taken for transcriptome analyses at 3.5 hours after IPTG-induction. To verify the occurrence of DNA uptake in this experiment, genomic DNA was added to the cells, as described before [17]. The transformation was monitored by plating cells on TY plates containing 2.5 µg ml^−1^ erythromycin. The transformation efficiency of the *comK_Bsu_* overexpression strain was comparable to that found in previous experiments (5–9 transformants/µg of genomic DNA). In all transformants the presence of erythromycin and chloramphenicol resistant markers was confirmed by PCR (data not shown).

**Figure 1:**
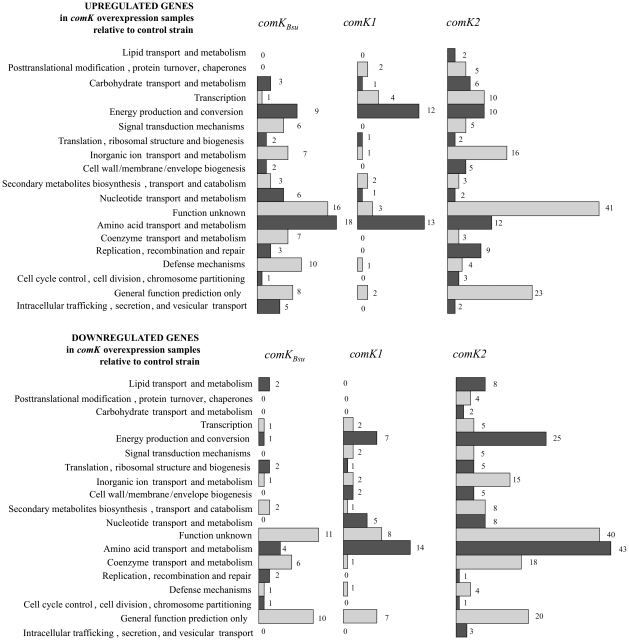
Numbers of genes belonging to the clusters containing commonly up- (above) or downregulated (bellow) genes upon *comK* overexpression classified using COG functional categories.

Differentially expressed genes in the late exponential phase are listed in Table S1. Genes known to be involved in DNA uptake and regulated by ComK in *B. subtilis* [7], [20] are listed in Table 1. In agreement with previous flow cytometric experiments [17], the most highly up-regulated genes were in the *comG* operon, encoding the DNA transport machinery. The upregulation of the *comGA* gene was also verified using quantitative RT-PCR on independent samples (81.9±17.4 times upregulation in *comK_Bsu_* expressing samples compared to the control strain). In addition, a stimulatory effect was observed for transcripts of the cytosolic proteins Smf, YwpH and RadC. Smf is required in *Streptococcus pneumoniae* to protect incoming transforming DNA [21], YwpH is probably a single-strand DNA binding protein [22], while RadC encodes a DNA repair protein. The induced expression of *comG* operon and genes involved in DNA recombination and repair was previously shown in *B. subtilis* when ComK level increased [7], [20]. However, other *com* genes that are known to be involved in competence development (e.g. the *comE*, *comF* operons and *comC* gene) did not show elevated expression levels similar to the genes of the *comG* operon (Table 1). The expression of *comEA* was followed using promoter-*gfp* constructs. The transcription driven from the *comEA* promoter was not altered in response to overexpression of *comK_Bsu_* and was comparable to the wild type cells without reporter constructs (Figure S1). Upon *comK_Bsu_* overexpression we also found many genes showing homology with genes unrelated to DNA uptake and recombination e.g. BC4679 (homolog of *B. subtilis* YcgA putative integral inner membrane protein), BC0497 (homolog of *B. subtilis* YfhF putative nucleotide binding protein) or BC1734 (homolog of *B. subtilis* YfiL putative ABC transporter, ATP-binding protein) (see Table S2). Genes belonging to the selected clusters were grouped into functional classes (Fig 1). Overrepresented clusters upon overexpression of *comK_Bsu_* gene contained the categories of amino acid transport and metabolism, energy production and conversion and defense mechanism.

**Table 1:** Transcriptional changes of the functional homologues of the *B. subtilis* DNA uptake apparatus in *B. cereus* ATCC15479 upon overexpression of *B. subtilis* ComK.

*Locus tag*	*B. subtilis competence-related protein*	*Description in SubtilList database*	*Ratio* a	*Significance (p-value)* b
BC1134	ComK (1)	Competence transcription factor	0.96	10^−1^
BC5250	ComK (2)	Competence transcription factor	1.3	10^−5^
BC1306	ComC	Prepilin peptidase	NA	NA
BC4324	ComEA	Exogenous DNA- binding protein	1.3	10^−5^
BC4323	ComEB	DNA binding and uptake	1.0	10^−1^
BC4322	ComEC	DNA binding and uptake	1.3	10^−3^
BC5193	ComFA	DNA binding and uptake	1.15	10^−2^
-	ComFB	Late competence gene	-	-
BC5192	ComFC	Late competence gene	1.3	10^−3^
BC4239	ComGA	Late competence gene	39.7	10^−15^
BC4238	ComGB	DNA transport machinery	29.9	10^−16^
BC4237	ComGC	Exogenous DNA-binding	39.0	10^−13^
BC4236	ComGD	DNA transport machinery	17.5	10^−6^
BC4235	ComGE	DNA transport machinery	NA	NA
BC4234	ComGF	DNA transport machinery	31.1	10^−16^
BC4233	ComGG	DNA transport machinery	NA	NA

a The ratio of gene expression is shown. Ratio: expression in the ComK_Bsu_ overexpressed samples over that in not ComK_Bsu_ overexpressed samples.

b Bayesian *p* value.

NA, no data are available (no probe are available for the gene).

These microarray data might explain the low efficiency of natural transformation in *B. cereus* ATCC 14579, because in the absence of e.g. ComEA, ComFA and NucA proteins the efficiency of natural transformation is reduced in *B. subtilis*, although still possible [23]–[26].

ComK*_Bsu_* was previously shown to bind *in vitro* to the promoter regions of both *comK* homologues of *B. cereus*, *comK*1 and *comK*2 [17]. In our microarray experiments, *comK1* showed no significant change in expression levels, while expression of *comK2* was slightly elevated (i.e. less than 2 fold). Thus, overexpression of *comK_Bsu_* results in elevated expression of selected genes coding for DNA uptake and recombination, but not of all genes described to be essential for efficient DNA uptake in *B. subtilis*.

### Transcriptional profiles of *comK1* and *comK2* overexpression strains

Since functional DNA uptake in *B. cereus* ATCC 14579 could be induced by overexpressing *comK_Bsu_*, we addressed the question whether the ComK_Bsu_ homologues ComK1 and ComK2 can induce expression of competence-related genes in *B. cereus*. Therefore, the *comK1* and *comK2* genes were separately cloned behind the *spac* promoter [27] that can be induced by isoporyl-β-D-thiogalactoside (IPTG) addition. The resulting plasmids pATK31 and pATK32, containing *comK1* and *comK2*, respectively, were introduced into *B. cereus* ATCC 14579 by electroporation, and an empty plasmid pLM5 was used as a control. The experiment was performed as described for ComK_Bsu_ overexpression. To test the occurrence of DNA uptake in these experiments, genomic DNA was added to the cells, but no transformants were observed. The analysis of the microarray data showed that *comK1* and *comK2* are responsible for activation of different sets of genes (see the 20 most up- or down-regulated genes in Table S2 and Table S3). Microarray results validated the overexpression of *comK1* and *comK2*, as the levels of both *comK1* and *comK2* mRNA were about 140 times enhanced in overexpressed strains for *comK1* and *comK2*. Unexpectedly, we did not find any genes related to DNA uptake or recombination. Upon *comK1* overexpression the most differentially expressed genes belonged to the functional categories representing amino acid transport and metabolism (e.g. BC1404, BC3317) and energy production and conversion (Fig 1). Interestingly, upon *comK2* overexpression we found mostly transcriptional regulators (e.g. BC4930, BC0958) and hypothetical proteins (e.g. BC5247, BC3399), but the same functional categories were also affected, e.g. amino acid transport and metabolism and energy production and conversion (Fig 1). Interestingly, we observed the upregulation of BC5251 when *comK2* was overexpressed in *B. cereus*. BC5251 codes for a RNA polymerase sigma factor in *B. cereus* ATCC 14579 and located downstream of *comK2* in reverse direction. The upregulation of BC5251 sigma factor in the *comK2* overexpression strains was also validated using quantitative RT-PCR experiments on independent samples. The BC5251 expression level was found to be more than 1000 times enhanced in the *comK2* overexpression samples compared to that of the wild type strains. Expression of BC5251 showed very weak changes when *comKBsu* or *comK1* was overexpressed (1.9±0.1 and 2.6±0.1, respectively) compared to *comK2* overexpression. This data suggests that the primary function of ComK1 and ComK2 in *B. cereus* might not be in competence development.

### P*comK1-gfp* and P*comK2-gfp* expression in wild type *B. cereus* and in *comK1* and *comK2* mutants

To get more insight into the effect of *B. cereus* ComK proteins on regulation of their own promoters and in the network between these two genes, we constructed fusions of *comK1* and *comK2* promoter regions with the *gfp* gene. The resulting plasmids, pILcomK1-gfp and pILcomK2-gfp, contain an in frame fusion of *gfp* with the first 6 codons of *B. cereus comK1* and *comK2*, respectively, and were used to determine the expression from these genes in the wild type strains under various growth conditions. Using strains grown in minimal medium (MM), a low signal of P*comK1*-*gfp* was detected compared to the wild type strain lacking the gfp construct, using flow cytometry analysis. In contrast, no *comK2* expression could be detected. The microarrays data, showed that overexpression of one of the *comK* genes did not alter the expression level of its paralog. To validate these results, we introduced *comK1* (pATK31) or *comK2* (pATK32) inducible constructs into strains harboring either pILcomK1-gfp or pILcomK2-gfp. In agreement with the microarray analysis, we did not observe any difference in the *gfp* expression for any of the promoters in the presence of ComK1 or ComK2 (data not shown).

**Figure 2:**
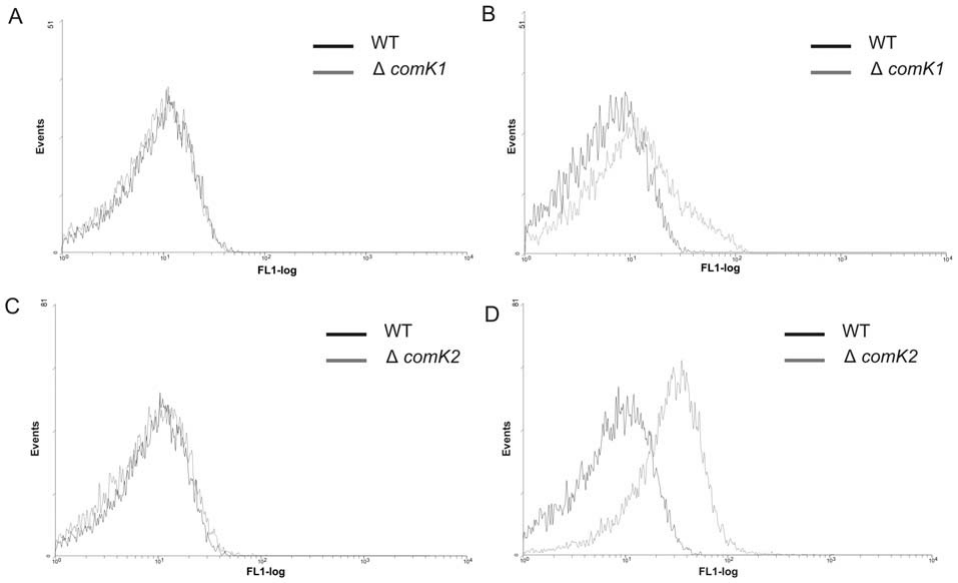
Flow cytometric analysis of PcomK1-gfp (A,C) and PcomK2-gfp (B,D) in liquid minimal medium. Promoter fusions in wild type (black) and in *comK1*(A,B) or *comK2*(C,D) mutant (gray). Analyses were performed as described in Experimental procedures. The numbers of cells are indicated on the *y* axis, and their relative fluorescence levels are indicated on the *x* axis on a logarithmic scale. For each experiment at least 20,000 cells were analysed. The graphs are the representative of at least three independent experiments.

Subsequently, we examined the effect of *comK1* and *comK2* mutations on the expression from the *comK* promoters. We replaced either *comK1* or *comK2* by a chloramphenicol cassette, resulting in strains Δ*comK1* and Δ*comK2*, respectively. First, we introduced pILcomK1-gfp and pILcomK2-gfp into the *ΔcomK1* strain and used the wild type *B. cereus* as a control containing the corresponding reporter constructs. While mutation of *comK1* did not alter the expression of P*comK1-gfp* in comparison to the wild type (Fig 2A), a higher P*comK2-gfp* expression was observed in the *comK1* mutant background (Fig 2B). This suggests that mutation in *comK1* affects only the expression of *comK2*, directly or indirectly, while it might not be involved in the regulation of its own transcription under the conditions examined.

We noticed that a mutation in the *comK2* gene did not alter the expression of P*comK1-gfp* (Fig 2C). In contrast, the *comK2* mutant harbouring pILcomK2-gfp showed increased *gfp* expression (Fig 2D), indicating that ComK2 might repress its own expression. Thus, while expression of the *comK1* gene seems to be independent of the presence or absence of the ComK1 or ComK2 protein, *comK2* expression depends on the presence of *comK* genes, but is not affected by the overexpression of *comK1* or *comK2* in the wild type cells.

### Expression of either *comK1* or *comK2* in the presence of ComK_Bsu_

**Figure 3:**
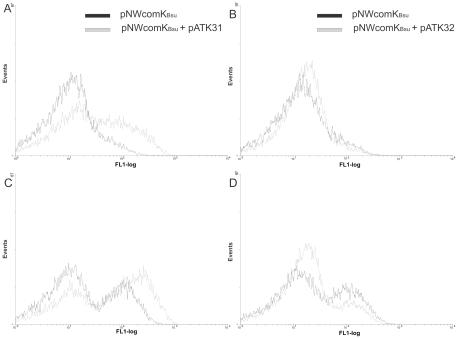
Flow cytometric analyses of PcomGA-gfp after overexpression of *comK_Bsu_* in the wild type containing pNWcomK_Bsu_ (black) and in the co-expressed *comK_Bsu_* and *comK1* (pATK31(A,C)) or c*omK2* (pATK32(B,C)) (gray). The samples were analyzed in three hours after IPTG induction; panels A,B- strains without induction, panels C,D- strains after induction with IPTG. The numbers of cells are indicated on the *y* axis, and their relative fluorescence levels are indicated on the *x* axis on a logarithmic scale. For each experiment at least 20,000 cells were analysed. The graphs are the representative of at least three independent experiments.

So far, we could only detect enhanced *comG* expression or low levels of transformation in *B. cereus*, when *comK_Bsu_* was overexpressed [17]. To examine whether ComK1 and/or ComK2 have an influence on this *comG* inducing effect of *comK_Bsu_* overexpression, we overexpressed either *comK1* or *comK2* in the presence of ComK_Bsu_ and monitored the effect on P*comGA-gfp* expression. First, we tested the effect of simultaneous *comK1* and *comK_Bsu_* expression on P*comGA-gfp* transcription. Strains grown in minimal medium were induced with 1 mM IPTG after reaching an OD_600_ of 0.75 and samples were taken for flow cytometric analysis every hour after induction. Overexpression of *comK1* and *comK_Bsu_* in minimal medium resulted in enhanced P*comGA-gfp* expression (Fig 3C and Table 2). It is noteworthy that non-induced samples showed also enhanced GFP levels (Fig 3A and Table 2). Most likely, this is due to the leakiness of the used promoters that was previously also reported [17], resulting in a small amount of protein that might activate P*comGA* transcription at a low level.

**Table 2:** Expression of the reporter gene (*gfp*) under different promoters.

Strains	PcomGA-gfp	PcomK1-gfp	PcomK2-gfp
ATCC 14579 (WT)	1.1±0.3	3.4±0.7	0.9±0.1
ATCC 14579 pNWcomK_Bsu_	26,6±1.9	ND	ND
ATCC 14579 pNWcomK_Bsu_ pATK31	42.0±3.0	ND	ND
ATCC 14579 pNWcomK_Bsu_ pATK32	16.8±2.7	ND	ND
ATCC 14579 Δ *comK1*	1.1±0.5	2.7±0.8	5.0±0.6
ATCC 14579 Δ *comK1* pNWcomK_Bsu_	8.2±0.7	ND	ND
ATCC 14579 Δ *comK2*	1.1±0.2	2.4±0.2	6.6± 2.7
ATCC 14579 Δ *comK2* pNWcomK_Bsu_	33.7±3.4	ND	ND

**Figure 4:**
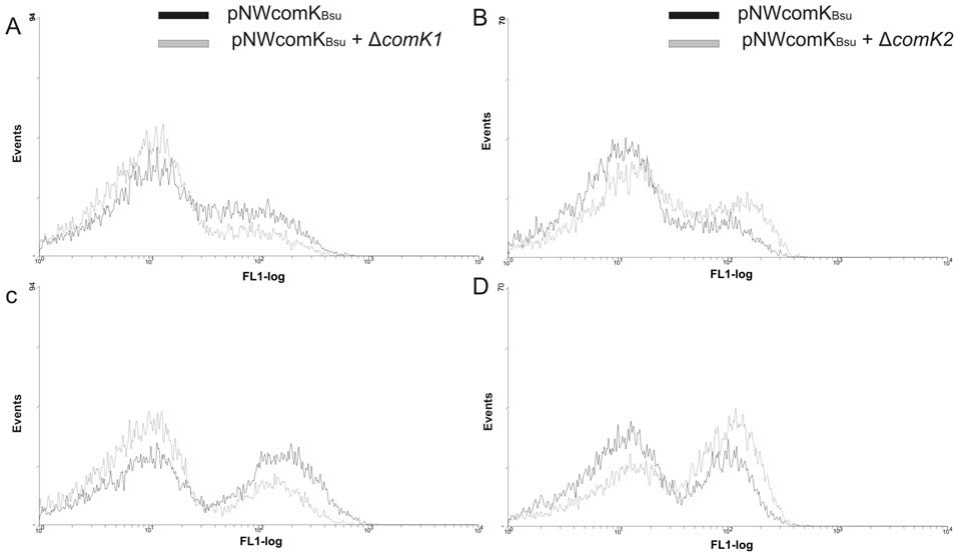
Single cell analyses of PcomGA-gfp and in liquid minimal medium. The effect of ComK_Bsu_ overexpression in the wild type (black) and in the *comK1* (A,C) or *comK2* (B,D) mutant (gray). The samples were analyzed in three hours after IPTG induction; panels A,B- strains without induction, panels C,D- strains after induction with IPTG. The numbers of cells are indicated on the *y* axis, and their relative fluorescence levels are indicated on the *x* axis on a logarithmic scale. For each experiment at least 20,000 cells were analysed. The graphs are the representative of at least three independent experiments.

Values are the geometric mean value of the whole population from the flow cytometric experiments (Figure 3–4) and given in arbitrary units with extracted background auto-fluorescence. Standard deviations are indicated.

A similar experiment for *comK2* overexpression was performed as described before for *comK1*. The transcription from P*comGA* in response to coexpression of *comK2* and *comK_Bsu_* was decreased in comparison to single induction of *comK_Bsu_* (Fig 3D and Table 2), suggesting a repressing role of ComK2. Notably, we could also detect a decreased P*comGA-gfp* expression in noninduced samples (Fig 3B and Table 2).

### ComK_Bsu_ overexpression in *comK1* and *comK2* mutants

The results above indicate that *comK1* and *comK2* might have opposite roles in the ComK_Bsu_ induced *comG* expression in *B. cereus*. To verify these data, we monitored the effect of the mutations in *comK1* and *comK2* genes on a P*comGA-gfp* expression in the presence of ComK_Bsu_.

Due to conflict in the applied antibiotic resistance markers of our constructs (i.e. both *comK* mutants and *comK_Bsu_* overexpression construct were constructed using *cat* resistance genes), we constructed a new *comK_Bsu_* inducible construct (pNWK-Km), where the kanamycin cassette was inserted into the chloramphenicol resistance gene. This *comK_Bsu_* overexpression construct (pNWK-Km) showed moderately increased *comG* expression compared to the original *comK_Bsu_* construct (pNWcomK_Bsu_) when cultures were not induced with IPTG. Changing the antibiotic resistance gene on the vector could cause differences in copy number or in the transcription activation on the plasmid, resulting in enhanced basal expression from the hyperspank promoter. After obtaining a *comK1* mutant strain containing pNWK-Km, the P*comGA-gfp* promoter fusion construct (pILcomGA-gfp) was subsequently introduced by electroporation. Strains were grown under the same condition as described before and the cells were induced with IPTG. In *comK1* mutant strain a lower P*comGA*-*gfp* expression was detected in the *comK_Bsu_* overexpression samples (Fig 4C and Table 2). This indicates in agreement with the overexpression constructs that ComK1 positively effects the expression of *comG* operon when *comK_Bsu_* is overexpressed in *B. cereus*.

Data on *comK2* overexpression suggested a negative effect of ComK2 on the expression of the *comG* gene in *comK_Bsu_* overexpressing *B. cereus*. As previously, the *comK2* mutant strain was subsequently transformed with pNWK-Km and pILcomGA-gfp constructs. In the *comK2* mutant background *comGA* transcription was increased, when *comK_Bsu_* was simultaneously overexpressed (Fig 4D and Table 2). Strikingly, expression from P*comGA* was detected at high levels in most of the cells in the population of the Δ*comK2* strain. Moreover the enhancing effect of the *comK2* mutation was observed in non-induced samples as well (Fig 4B and Table 2). The small amount of ComK_Bsu_ that is produced by basal expression from the promoter might be able to activate transcription at P*comGA* more efficient in the absence of ComK2. Taken together, these data suggest that ComK2 has a negative effect on the expression of the competence-related gene *comGA* in *B. cereus* in combination with *comK_Bsu_* overexpression.

To support our observations, we examined the effect of *comK1* or *comK2* deletion on the expression of *comEA* gene in *B. cereus*. Deletion of neither *comK1* nor *comK2* changed the expression pattern of *comEA* gene in *B. cereus* when *comK_Bsu_* was overexpressed, suggesting that the effect of *comK1* or *comK2* mutations on ComK_Bsu_ dependent *comGA* induction is specific (Figure S1).

## Discussion

Regulation of DNA uptake and recombination is achieved in various ways in bacteria. Within the *Bacillus* genus natural competence has been shown to be activated by the transcription factor ComK [5]. So far, only a limited number of *Bacillus* sp. has been shown to have the ability of reaching high efficient natural competence, such as *B. subtilis*, *B licheniformis* and *B. amiloliquefaciens* [28]–[30]. However, only a limited number of strains within a species show this phenotype under laboratory conditions, while in other cases to achieve high transformability the protein level of the ComK transcription factor should be increased by overexpression of its own *comK* gene [18], [19] or by disrupting the degradation of the ComK protein [31]. We have used similar methods previously to show the presence of functional DNA uptake in *B. cereus* ATCC 14579 [17]. However, we achieved a low-efficient DNA uptake induction only by overexpression of the heterologous *comK_Bsu_*. In this study we used various molecular methods to follow the effect of different *comK* species overexpression in *B. cereus*. We followed the effect of *comK_Bsu_*, *comK1* and *comK2* overexpression in *B. cereus* using microarray techniques and showed that competence-related genes are induced only when the *comK_Bsu_* was overexpressed. However, only part of the competence-related genes is activated, while *comK1* expression was not changed and *comK2* showed a slightly increased level of expression in the *B. cereus* strain containing overexpressed *comK_Bsu_*. As shown before, ComK_Bsu_ binds to the promoter regions of several late competence genes and *comK* genes of *B. cereus* [17]. In agreement with flow cytometric analysis the *comG* operon in the *comK_Bsu_* overexpression strain was highly up-regulated, while we did not notice significantly enhanced expression of the *comE* or *comF* operons. This implies that ComK_Bsu_ might not activate transcription of these genes *in vivo* although ComK_Bsu_ was shown to bind to the *comE*, *comK1* and *comK2* promoter fragments *in vitro* [17]. The conflict between the *in vivo* and *in vitro* observation might originate from the position of the ComK binding site relative to the promoter -35 and -10 sites. Transcription activation by ComK_Bsu_ is helix face dependent as a 6-bp insertion between the ComK box and -35 hexamer of the *B. subtilis comG* promoter abolished activation of transcription [32]. On the other hand, we could not identify the so-called K-box (ComK_Bsu_ binding site) in any of the *B. cereus* genes coding for the homologs of the DNA uptake apparatus. Interestingly, other competence-related genes involved in DNA binding or recombination, like *radC* and *ywpH*, showed an enhanced level of expression when *comK_Bsu_* was overexpressed. The lack of high induction of the complete set of competence-related genes in the *comK_Bsu_* overexpressing *B. cereus* strain might explain the observed low transformability of *B. cereus* under the conditions used. Previous studies on *B. subtilis* showed that when not all the genes for the competence machinery are functional, transformation is still possible, though the efficiency is much lower (e.g. Δ*comE* [23]).

In contrast to *comK_Bsu_* overexpression, increasing the level of *comK1* or *comK2* alone, activated different sets of genes unrelated to DNA uptake. We noticed altered expression of more than 100 genes by *comK1* overexpression, while more than 300 genes have altered gene expression in the *comK2* overexpressing *B. cereus*, and several of these genes were located in operons. The lack of altered gene expression related to DNA uptake could be the result of overexpression of *comK1* and *comK2* separately or because the target genes of these regulators are different under the conditions used. It is also possible that ComK1 and ComK2 have other primary functions in *B. cereus* than modulating the expression of genes related to DNA uptake and recombination. This is also supported by the increased transcription of a σ-factor (BC5251) located adjacent to *comK2* when *comK2* was overexpressed in *B. cereus*. Interestingly, upon overexpression of *comK_Bsu_*, *comK1* or *comK2* in *B. cereus*, similar functional categories (e.g. amino acid transport and metabolism, energy production and conversion) were overrepresented (Fig 1), although the list of genes was not overlapping. The connection between regulation of competence related genes and amino acid metabolism is not unprecedented, e.g. *B. subtilis* CodY which, next to its major function as branched-chain amino acid metabolism regulator, modulates competence development in *B. subtilis* [33]. Strikingly, although similar functional categories are overrepresented in the microarray experiments, we did not find common genes upregulated upon *comK1* and *comK2* overexpression, suggesting highly different regulon for these two *comK* homologues. The relatively high level of mRNA level increase in the induced overexpression strains suggest a very low or almost absent expression of *comK* genes in wild type cells.

The target genes of ComK1 and ComK2 are different in the overexpression strains which shows the divergence of the two ComK proteins in *B. cereus*. Although both ComK1 and ComK2 show conserved regions homologous to ComK_Bsu_, the ComK2 protein lacks the 22 aminoacids long C-terminal region [5]. Interestingly, deletion of the 25 aminoacids C-terminal part disrupted the ability of ComK_Bsu_ to activate transcription on the *comGA* promoter *in vivo*, but preserved its DNA binding ability [34]. One could hypothesize that ComK2 represses genes, like *comGA* transcription, in its short form, but once was able to activate genes, like *comGA* in an ancient longer form (without deletion at the C-terminus) of the protein, which lost these amino acids during evolution.

It will be interesting to investigate the target genes and promoters found in the *comK1* and *comK2* overexpression studies and perform EMSA experiments to see if the genes identified by transcriptomics are directly or indirectly regulated by the different ComK proteins and to define a DNA binding site for both ComK proteins of *B. cereus*. However, in this study we concentrated on the ability of ComK1 and ComK2 to modulate the expression of competence-related genes and other genes. Interestingly, we show that overexpression of *comK1* or *comK2* in the presence of ComK_Bsu_ results in changed activation of P*comGA-gfp*. The simultaneous overexpression of *comK1* and *comK_Bsu_* resulted in enhanced expression from P*comGA-gfp* compared to single induction of *comK_Bsu_*, while deletion of *comK1* reduced the effect of *comK_Bsu_* overexpression on the c*omGA* expression.

In contrast to *comK1*, the overexpression of *comK2* or deletion of *comK2* in the presence of ComK_Bsu_ resulted in reduced or increased *comGA* expression in *B. cereus*, respectively.

The overexpression or deletion of *comK1* and *comK2* genes modulate the ComK_Bsu_ induced *comG* transcription, but has no effect on *comG* expression in the absence of the ComK_Bsu_ protein. It is therefore possible that *B. cereus* ATCC 14579, if it is a naturally competent bacterium under specific conditions, has a different regulatory mechanism than that of the model organism *B. subtilis*. This view is also supported by the observation that the upstream regulatory pathway is less conserved in *Bacilli* [5].

Taken together, we propose that ComK1 and ComK2 take an opposite role on the modulation of the ComK_Bsu_ effect in *B. cereus*. Future studies should reveal the functions of ComK1 and ComK2, and whether any protein-protein interaction exists between the ComK proteins, how ComK1 and ComK2 proteins activate or repress transcription *in vivo* and *in vitro*, and if they compete for DNA binding sites at target promoters.

## Methods

### Bacterial strains and media

The strains and plasmids used in this study are listed in Table 3. *B. cereus* strains were grown in TY (10 gL^−1^ trypton, 5 gL^−1^ yeast extract, 5 gL^−1^ NaCl, 0.1 mM MnCl_2_) or in minimal medium MM (62 mM K_2_HPO_4_, 44 mM KH_2_PO_4_, 15 mM (NH_4_)_2_SO_4_, 5.6 mM sodium citrate, 0.8 mM MgSO_4_, 0,02% of casamino acids, 27.8 mM glucose and growth factors [35]. Growth factors were made by adding tyrosine, tryptophan, methionine, histidine, adenine, uracil (final concentration 20 µg/ml), nicotinic acid and riboflavin (final concentration 0.5 µg/ml) to water. For cloning, *Escherichia coli* MC1061 and *Lactococcus lactis* MG1363 were grown in TY and GM17 (37.5 gL^−1^ M17 broth (Difco), 0.5% glucose) broth medium, respectively. Bacterial strains were grown at 30°C or 37°C, supplemented with appropriate antibiotics, erythromycin (5 µg ml^−1^), chloramphenicol (3–5 µg ml^−1^) or kanamycin (50 µg ml^−1^).

**Table 3:** Strains and plasmids used in the study.

Strains and plasmids	Relevant characteristics/plasmids	Reference
***E. coli***		
HB101	pRK24; strain for conjugation	[41]
MC1061	*F- araD139 Δ(ara-leu) 7696 galE15 galK16 Δ(lac)X74 rpsL (Strr) hsdR2 (rK_ mK+) mcrA mcrB1*	Laboratory stock
***L. lactis***		
MG1363	*Lac^–^ Prt^–^;* plasmid-free derivative of NCDO712	[45]
***B. cereus***		
ATCC 14579	type strain	BGSC
ATCC 14579	pATK31, pNWcomK_Bsu_, pILcomGA-gfp	This study
ATCC 14579	pATK32, pNWcomK_Bsu_, pILcomGA-gfp	This study
ATCC 14579	pNWcomK_Bsu_, pILcomGA-gfp	This study
ATCC 14579	pNWK-km_u_, pILcomEA-gfp	This study
ATCC 14579 Δ*comK1*	*comK1:: cm*	This study
ATCC 14579 Δ*comK1*	pILcomK1-gfp	This study
ATCC 14579 Δ*comK1*	pILcomK2-gfp	This study
ATCC 14579 Δ*comK1*	pNWK-km, pILcomGA-gfp	This study
ATCC 14579 Δ*comK1*	pNWK-km, pILcomEA-gfp	This study
ATCC 14579 Δ*comK2*	*comK2:: cm*	This study
ATCC 14579 Δ*comK2*	pILcomK1-gfp	This study
ATCC 14579 Δ*comK2*	pILcomK2-gfp	This study
ATCC 14579 Δ*comK2*	pNWK-Km, pILcomGA-gfp	This study
ATCC 14579 Δ*comK2*	pNWK-Km, pILcomEA-gfp	This study
***plasmids***		
pNWcomK_Bsu_	pNW33N containing *B. subtilis comK*	[17]
pNW33N	*Geobacillus-Bacillus-E. coli* shuttle vector, Cm^R^	Genetic Stock Center
pIL253	*ermAM, ery^r^*	[46]
pSG1151	*amp^r^, cat*	[43]
pcomK1-gfp	pSG1151 containing P*comK1* fused to *gfp*	This study
pcomK2-gfp	pSG1151 containing P*comK2* fused to *gfp*	This study
pcomEA-gfp	pSG1151 containing P*comEA* fused to *gfp*	This study
pILcomK1-gfp	pIL253 containing P*comK1-gfp*	This study
pILcomK2-gfp	pIL253 containing P*comK2-gfp*	This study
pILcomEA-gfp	pIL253 containing P*comEA-gfp*	This study
pILcomGA-gfp	pIL253 containing P*comGA-gfp*	[17]
pNWK-Km	pNWcomK_Bsu_ containing km^R^ cassette	This study
pLM5	Vector cointaining spac promoter and lacI repressor, km^R^	[27]
pATK31	pLM5 containing *B. cereus comK1*	This study
pATK32	pLM5 containing *B. cereus comK2*	This study
pUC19C	pUC19 vector containing *cat^R^*	Laboratory stock
pBlueScript SK	*amp^R^*	Stratagene
pATΔS28	*tra*_ conjugative suicide vector for *B. cereus* group, Spc^R^	[41]
pBtcomK1	pBtSK, contating comK1 region with cat^R^ casette	This study
pATΔcomK1	pATΔS28, containing comK1 region with cat^R^ cassette	This study
pBtcomK2	pBtSK, contating comK2 region with cat^R^ casette	This study
pATΔcomK2	pATΔS28, containing comK2 region with cat^R^ cassette	This study

### RNA isolation, preparation of labeled cDNA, and hybridization

Cells were grown overnight in 10 ml of TY medium supplemented with chloramphenicol (5 µg ml^−1^) or kanamycin (50 µg ml^−1^) for *comK_Bsu_* or *comK1/comK2* overexpression, respectively. Next, the cultures were diluted to an OD_600_ of 0.15 in 25 ml of minimal medium containing appropriate antibiotics. Samples for transcriptome analyses were induced at the exponential-growth phase (OD_600_≈0.75) with isopropyl-β-D-thiogalactopyranoside (IPTG) to a final concentration of 1 mM. Cells were harvested 3 h after induction. Three independent cultures of each strain were used. RNA was isolated from 15 ml of culture, as described previously [36]. RNA was eluted with 60 µl of elution buffer. A total amount of 20 µg RNA was used for the reverse transcriptase reaction with SuperscriptIII (Gibco BRL). DNA-microarrays containing amplicons of 5200 annotated genes in the genome of *B. cereus* ATCC 14579 were designed and produced as described previously [37]. Slide spotting, slide treatment after spotting, and slide quality control were performed as described elsewhere. Data were analyzed essentially as described before [38]. Each ORF is represented by duplicate spots on the array. After hybridization, fluorescent signals were quantified with the ArrayPro analyzer, and processed with Micro- Prep [37]. Statistical analysis was performed using CyberT [39]. Genes with a Bayes P-value below 1.0×10^−4^ with at least twofold differential expression were considered to be significantly affected. Microarrays data is MIAME compliant and that the raw data has been deposited in a MIAME compliant Gene Expression Omnibus database (GSE27267), as detailed on the MGED Society website http://www.mged.org/Workgroups/MIAME/miame.html.

### Quantitative RT-PCR

Samples obtained as described above for the microarray experiments were treated with RNase-free DNase I (Fermentas, St. Leon-Rot, Germany) for 60 min at 37°C in DNaseI buffer (10 mmol·l^−1^ Tris·HCl (pH 7.5), 2.5 mmol·l^−1^ MgCl_2_, 0.1 mmol·l^−1^ CaCl_2_). Samples were purified with the Roche RNA isolation Kit. Reverse transcription was performed with 50 pmol random nonamers on 4 µg of total RNA using RevertAid™ H Minus M-MuLV Reverse Transcriptase (Fermentas, St. Leon-Rot, Germany). Quantification of cDNA was performed on an CFX96 Real-Time PCR System (BioRad, Hercules, CA) using Maxima SYBR Green qPCR Master Mix (Fermentas, St. Leon-Rot, Germany). The following primers were used: for BC4239, qBCE9 and qBCE10, for BC5251, qBCE19 and qBCE20 and for *rpoA* gene of *B. cereus*, qBCE3 and qBCE4 (primer sequences are listed in Table 4). The amount of BC0753 cDNA was normalized to the level of *rpoA* cDNA using the 2^−ΔΔCt^ method [40].

**Table 4:** Oligonucleotides used in this study.

Oligonucloetides	Sequence (5′-3′)
oAM-9	TTGTCCATAGTATACAGCTCCTTT
oAM-11	CGGAGCTCTGTTATTTGTGGGCAGTAAGAC
oAM-12	CAACATTTATGAGCGCTGACGGTGTA
oAM17-b	CGCCCCGGGCACTAAAGGGAACAAAAG
oAM18	CGTCTAGAACTATAGGGCGAATTGGAG
oAM-20	CATGGCATGCGGGTCTTACTGCCCACAAA
oAM-21	GCGGAAGCTTAAGGGAGGACTTAAAATCAT
oAM-22	CATGGCATGCATCGGCAAGCACTCGAGAAA
comK1-*Apa*I-F	CAGGGGCCCGGTTTAGCAAAGAATAGC
comK1-*Eco*RI-R	CGCGAATTCCCACTTTACTTTCCATAC
pEA-*Apa*I-F	GCACGGGCCCTGTGATTGTTCGATTAC
pEA-*Eco*RI-R	GCCGGAATTCATCCCACATCATTTTAC
comK2-*Apa*I-F	CGCGGGCCCTTTCGCTTCGTATAATG
comK2-*Eco*RI-R	CGCGAATTCTTTCATCATTCATGATTTT
K1-F	CGCGCGAGCTCACAGCTCCTTTTTGG
K1-R	CGCGAAGCTTGCGCTGACGGTGTAAACG
K2-S-F	CGCGCATGCAATCGATGCACAAGTC
K2-E-R	CGCGAATTCTGGCTCTTCCAAGAGTC
qBCE3	CGTGGATATGGTACTACTTTGG
qBCE4	TTCTACTACGCCCTCAACTG
qBCE9	ATCAAGAAAGTCTCGTTATTCGCC
qBCE10	GTCCAGTAAACACAAGTAGCCC
qBCE19	CAATGTGGTTTAATCGGTCTTTGG
qBCE20	TTCTCCTTCTCATCTAACACGCT

### Construction of pATK31 and pATK32

To overexpress ComK homologues the *comK*1 and *comK*2 genes were amplified with oAM19 and oAM20, oAM21 and oAM22, respectively. PCR products were cloned into the Eco47III site of pLM5 vector [27], resulting in pATK31 and pATK32, respectively. Plasmids were introduced into *B. cereus* ATCC 14579 by electroporation. IPTG was used at a final concentration of 1 mM to induce the overproduction of proteins.

### Construction of a *comK*1 and *comK*2 null mutant

First, the *comK1* region was amplified from genomic DNA of *B. cereus* ATCC15479 with primers oAM-9 and oAM-12. The PCR product was cloned into pBtSK digested with HincII and ScaI, resulting in pBtSKcomK1. Subsequently, the *comK1* gene was cut out with XbaI and EcoRV and replaced by a chloramphenicol cassette from pUC19C. Finally, the insert containing *comK1* upstream and downstream flanking regions with chloramphenicol cassette was amplified from the vector with primers K1-F and K1-R. Subsequently, this fragment was cloned into pATΔS28, resulting in pATΔcomK1.

To knockout the *comK2* gene, first the *comK2* region was amplified with primers K2-S-F and K2-E-R. The resulting fragment was cloned into the pBtSK vector digested with ApaI and XbaI and blunted by Klenow polymerase. Next, *comK2* was cut out with XhoI and SfuI and replaced by a chloramphenicol cassette from pUC19C. The resulting plasmid pcomK2_cm was digested with PuvII and the insert containing *comK2* upstream and downstream flanking regions with a chloramphenicol cassette was cloned into pATΔS28 [41], resulting in pATΔcomK2. The orientation of the inserts in the vectors was checked by restriction analysis.

The vectors were then transformed into *E.coli* HB101/pRK24 and the resulting strains were used in conjugation experiments with *B. cereus*. Conjugation was performed as described by Trieu-Cuot [42]. Transconjugants were selected for chloramphenicol resistance and spectinomycin sensivity. PCR and Southern analysis confirmed that the strain harbored the deleted allele of *comK1* and *comK2* and that the chloramphenicol resistance cassette had recombined into the chromosome through a double-crossover event (data not shown).

### Construction of the pILcomK1-gfp, pILcomK2-gfp and pILcomEA-gfp vectors

The *comK1*, *comK2* and *comEA* promoter regions, including the ribosome binding site, were amplified by PCR using primers comK1-*Apa*I-F and comK1-*Eco*RI-R for P*comK*1, comK2-*Apa*I-F and comK2-*Eco*RI-R for P*comK*2 and pEA-*Apa*I-F and pEA-*Eco*RI-R for P*comEA*, respectively. After digesting with *Eco*RI and *Apa*I the PCR products were ligated into the corresponding sites of pSG1151 [43], resulting in pcomK1-gfp, pcomK2-gfp and pcomEA-gfp vectors, respectively. These plasmids were used as a template to amplify P*comK1-gfp*, P*comK2-gfp* and P*comEA-gfp* by PCR using primers oAM17-b and oAM18. The resulting PCR fragments were digested with *Xba*I and *Eco*47III, and inserted into *Xba*I-*Sma*I cleaved pIL253 and introduced into *L. lactis* MG1363 by electroporation [44]. The correct cloned DNA sequence was confirmed by sequencing. Subsequently, plasmids pILcomK1-gfp, pILcomK2-gfp and pILcomEA-gfp were introduced into the wild type, *B.cereus* Δ*comK1*, *B. cereus* Δ*comK2* by electroporation.

### Analysis of reporter gene expression

For flow cytometric analyses *B. cereus* ATCC 14579 and *B. cereus* Δ*comK1* and Δ*comK2* strains carrying either pILcomGA-gfp, pILcomK1-gfp, pILcomK2-gfp or pILcomEA-gfp were grown ON in TY supplemented with erythromycin (5 µg ml^−1^) and chloramphenicol (5 µg ml^−1^) for the mutants strains. For the flow cytometric analyses, cultures were inoculated into fresh minimal medium with erythromycin (2.5 µg ml^−1^ in MM) and chloramphenicol (5 µg ml^−1^) for *comK* mutants After transition point, samples were taken every hour.

Cells were diluted in minimal salts and analyzed on a Coulter Epics XL-MCL flow cytometer (Beckman Coulter Mijdrecht, NL) operating an argon laser at 488 nm. Green fluorescent protein (GFP) signals were collected through an FITC filter with the photomultiplier voltage set between 700 and 800 V. Date were obtained using EXPO32 software (Beckman Coulter) and further analyzed using WinMDI 2.8 (The Scripps Research Institute). Figures were prepared using WinMDI 2.8 and CorelDraw X3 (Corel Corporation).

For the plate reader experiments, *B. cereus* cells containing either pILcomGA-gfp or pILcomEA-gfp were grown as described above. The OD and fluorescence were measured every 15 minutes using a TECAN F200 Microplate Reader (TECAN Group Ltd, Mannedorf, Switzerland). Obtained fluorescence data from at least 3 independent experiment was normalized to OD and given in arbitrary units.

## Supporting Information

Figure S1 **Level of green fluorescent protein (gfp) in** ***B. cereus*** **cells carrying the pNWK-Km** ***comK_Bsu_*** **overexpression plasmid and pILcomGA-gfp (triangle) or pILcomEA-gfp (circle in wild type, square in** ***comK1*** **deletion and rhombus in** ***comK2*** **deletion strains) reporter plasmids.** (−) Strains without induction of *comK_Bsu_* overexpression are indicated with open symbol, while (+) strains with *comK_Bsu_* induction are denoted with filled symbols. Fluorescence of wild type cells without any reporter constructs (cross). OD and fluorescence was measured every 15 minutes using a TECAN F200 Microplate Reader. Obtained fluorescence data from 3 independent experiments were normalized to OD and given in arbitrary units. Time is indicated on the *y* axis in seconds and fluorescence in arbitrary units is given on the *x* axis.
(PDF)

Table S1 **Summary of transcriptional changes in** ***B. cereus*** **ATCC15479 upon overexpression of** ***B. subtilis comK*** **unrelated to DNA uptake.** The top 20 genes significantly up-regulated are shown in the table. The complete list of transcriptional changes is available at the Gene Expression Omnibus database under the accession number GSE27267. ^a^ The ratio of gene expression is shown. Ratio: expression in the *comK_Bsu_* overexpressed samples over control samples. ^b^ Bayesian *p* value.
(PDF)

Table S2 **Summary of transcriptional changes in** ***B. cereus A*** **TCC15479 upon overexpression of** ***comK1*** **.** The top 20 genes significantly up- or down-regulated are shown in the table. The complete list of transcriptional changes is available at the Gene Expression Omnibus database under the accession number GSE27267. ^a^ The ratio of gene expression is shown. Ratio: expression in the *comK2* overexpressed samples over control samples. ^b^ Bayesian *p* value.
(PDF)

Table S3 **Summary of transcriptional changes in** ***B. cereus*** **ATCC15479 upon overexpression of** ***comK2*** **.** The top 20 genes significantly up- or down-regulated are shown in the table. The complete list of transcriptional changes is available at the Gene Expression Omnibus database under the accession number GSE27267. ^a^ The ratio of gene expression is shown. Ratio: expression in the *comK2* overexpressed samples over control samples. ^b^ Bayesian *p* value.
(PDF)
