# Supplementary material for: Distinct Roles of ComK1 and ComK2 in Gene Regulation in Bacillus cereus
Source: PLoS One. 2011 Jul 1;6(7):e21859. doi: 10.1371/journal.pone.0021859 (PMC3128618; doi:10.1371/journal.pone.0021859)

Figure S1

Level of green fluorescent protein (gfp) in *B. cereus* cells carrying the pNWK-Km $comK_{Bsu}$ overexpression plasmid and pILcomGA-gfp (triangle) or pILcomEA-gfp (circle in wild type, square in *comK1* deletion and rhombus in *comK2* deletion strains) reporter plasmids. (-) Strains without induction of $comK_{Bsu}$ overexpression are indicated with open symbol, while (+) strains with $comK_{Bsu}$ induction are denoted with filled symbols. Fluorescence of wild type cells without any reporter constructs (cross). OD and fluorescence was measured every 15 minutes using a TECAN F200 Microplate Reader. Obtained fluorescence data from 3 independent experiments were normalized to OD and given in arbitrary units. Time is indicated on the *y* axis in seconds and fluorescence in arbitrary units is given on the *x* axis.

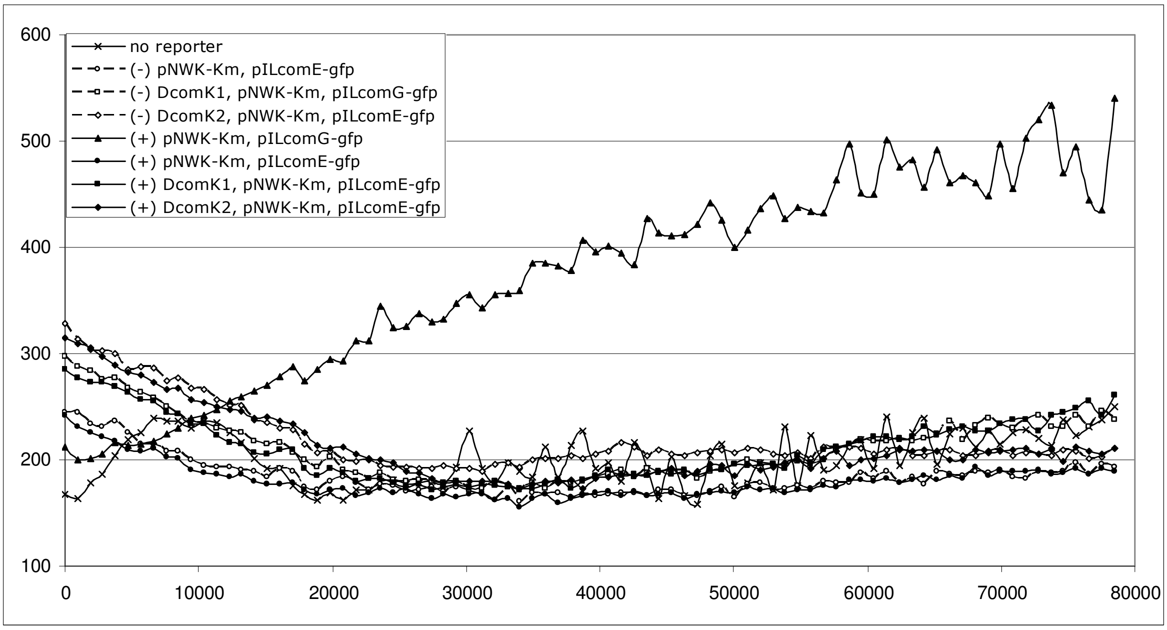

Supplement: Figure S1 — Level of green fluorescent protein (gfp) in B. cereus cells carrying the pNWK-Km comKBsu overexpression plasmid and pILcomGA-gfp (triangle) or pILcomEA-gfp (circle in wild type, square in comK1 deletion and rhombus in comK2 deletion strains) reporter plasmids. (−) Strains without induction of comKBsu overexpression are indicated with open symbol, while (+) strains with comKBsu induction are denoted with filled symbols. Fluorescence of wild type cells without any reporter constructs (cross). OD and fluorescence was measured every 15 minutes using a TECAN F200 Microplate Reader. Obtained fluorescence data from 3 independent experiments were normalized to OD and given in arbitrary units. Time is indicated on the y axis in seconds and fluorescence in arbitrary units is given on the x axis. (PDF) [file pone.0021859.s001.pdf]
