# Supplementary material for: Distinct Roles of ComK1 and ComK2 in Gene Regulation in Bacillus cereus
Source: PLoS One. 2011 Jul 1;6(7):e21859. doi: 10.1371/journal.pone.0021859 (PMC3128618; doi:10.1371/journal.pone.0021859)
Supplement: Table S1 — Summary of transcriptional changes in B. cereus ATCC15479 upon overexpression of B. subtilis comK unrelated to DNA uptake. The top 20 genes significantly up-regulated are shown in the table. The complete list of transcriptional changes is available at the Gene Expression Omnibus database under the accession number GSE27267. a The ratio of gene expression is shown. Ratio: expression in the comKBsu overexpressed samples over control samples. b Bayesian p value. (PDF) [file pone.0021859.s002.pdf]

Table S1. Summary of transcriptional changes in *B. cereus* ATCC15479 upon overexpression of *B. subtilis comK* unrelated to DNA uptake. The top 20 genes significantly up-regulated are shown in the table. The complete list of transcriptional changes is available at the Gene Expression Omnibus database under the accession number GSE27267. [a] The ratio of gene expression is shown. Ratio: expression in the $comK_{Bsu}$ overexpressed samples over control samples. [b] Bayesian *p* value

Locus tag	*B. subtilis homologue*	*Description in SubtilList database*	*Ratio*[a]	*Significance (p-value)*[b]
BC4679	YcgA	putative integral inner membrane protein	30,0	10^{-13}
BC0497	YfhF	putative nucleotide binding protein	25,3	10^{-12}
BC0496	PyrC	dihydroorotase	27,4	10^{-13}
BC4630	ArgG	argininosuccinate synthase	26,3	10^{-13}
BC4629	ArgH	argininosuccinate lyase	25,9	10^{-11}
BC1822	Pdp	pyrimidine-nucleoside phosphorylase	20,7	10^{-11}
BC1821	NupC	pyrimidine-nucleoside Na+(H+) cotransporter	20,4	10^{-11}
BC1374	YhaA	putative amidohydrolase	16,6	10^{-15}
BC4462	Maf	maf like protein	16,3	10^{-11}
BC1734	YfiL	putative ABC transporter ATP-binding protein	12,6	10^{-11}
BC4461	adC	DNA repair protein	11,5	10^{-14}
BC1542	PanD	aspartate alpha-decarboxylase	9,6	10^{-12}
BC1541	PanC	pantoate--beta-alanine ligase	8,1	10^{-15}
BC1966	ThrB	homoserine kinase	7,8	10^{-15}
BC2698	YwqG	hypothetical protein	5,4	10^{-12}
BC1965	ThrC	threonine synthase	5,1	10^{-11}
BC4130	ArgC	N-acetyl-gamma-glutamyl-phosphate reductase	4,8	10^{-11}
BC1820	DeoC	deoxyribose-phosphate aldolase	4,5	10^{-11}
BC2907	YueF	putative integral inner membrane protein	4,4	10^{-11}
BC2154	YwpH	Single-strand DNA binding- protein	4,2	10^{-11}
