# Supplementary material for: Distinct Roles of ComK1 and ComK2 in Gene Regulation in Bacillus cereus
Source: PLoS One. 2011 Jul 1;6(7):e21859. doi: 10.1371/journal.pone.0021859 (PMC3128618; doi:10.1371/journal.pone.0021859)
Supplement: Table S2 — Summary of transcriptional changes in B. cereus A TCC15479 upon overexpression of comK1 . The top 20 genes significantly up- or down-regulated are shown in the table. The complete list of transcriptional changes is available at the Gene Expression Omnibus database under the accession number GSE27267. a The ratio of gene expression is shown. Ratio: expression in the comK2 overexpressed samples over control samples. b Bayesian p value. (PDF) [file pone.0021859.s003.pdf]

Table S2 Summary of transcriptional changes in *B. cereus A*TCC15479 upon overexpression of *comK1*. The top 20 genes significantly up- or down-regulated are shown in the table. The complete list of transcriptional changes is available at the Gene Expression Omnibus database under the accession number GSE27267. [a] The ratio of gene expression is shown. Ratio: expression in the *comK2* overexpressed samples over control samples. [b] Bayesian *p* value

Putative ComK1-upregulated genes.

Locus tag	*Expression ratio*[a]	*Significance p-value*[b]	*Annotation*[c]
**Upregulated**			
BC1134	139.5	10^{-10}	Competence transcription factor (*comK1*)
BC0629	70.6	10^{-9}	Arginine/ornithine antiporter
BC0171	34.7	10^{-8}	Chitooligosaccharide deacetylase
BC3317	18.4	10^{-7}	Histidyl-tRNA synthetase
BC1407	11.5	10^{-7}	Imidazoleglycerol-phosphate dehydratase
BC1405	11.0	10^{-7}	ATP phosphoribosyltransferase
BC1404	10.5	10^{-6}	Histidyl-tRNA synthetase
BC1408	10.0	10^{-6}	Amidotransferase hisH
BC1411	9.1	10^{-6}	Phosphoribosyl-AMP cyclohydrolas
BC1409	8.8	10^{-6}	Phosphoribosylformimino-5-aminoimidazole carboxamide ribotide isomerase
BC1413	8.7	10^{-6}	Histidinol-phosphatase
BC4159	8.6	10^{-6}	2-oxoisovalerate dehydrogenase alpha subunit
BC4157	8.3	10^{-6}	Lipoamide acyltransferase component of branched-chain alpha-keto acid dehydrogenase complex
BC1410	8.0	10^{-6}	HisF protein, imidazole glycerol phosphate synthase subunit
BC4158	7.7	10^{-6}	2-oxoisovalerate dehydrogenase beta subunit
BC1406	7.3	10^{-6}	Histidinol dehydrogenase
BC4160	7.2	10^{-6}	Dihydrolipoamide dehydrogenase
BC4161	4.7	10^{-5}	Branched-chain-fatty-acid kinase
BC1412	4.7	10^{-5}	Phosphoribosyl-ATP pyrophosphatase
BC0102	1.7	10^{-5}	Negative regulator of genetic competence clpC/mecB

Putative ComK1-downregulated genes.

Locus tag	*Expression ratio*[a]	*Significance p-value*[b]	*Annotation*[c]
**Downregulated**			
BC3652	0.01	10^{-7}	Histidine ammonia-lyase
BC3651	0.01	10^{-7}	Urocanate hydratase
BC4126	0.03	10^{-6}	Ornithine carbamoyltransferase
BC4128	0.04	10^{-6}	Acetylglutamate kinase
BC4127	0.04	10^{-6}	Acetylornithine aminotransferase
BC4148	0.06	10^{-6}	Arginine transport ATP-binding protein artP
BC4129	0.07	10^{-5}	Glutamate N-acetyltransferase
BC4629	0.07	10^{-5}	Argininosuccinate lyase
BC4630	0.07	10^{-5}	Argininosuccinate synthase
BC4149	0.09	10^{-5}	Arginine transport system permease protein artQ
BC4131	0.09	10^{-5}	hypothetical protein
BC3886	0.1	10^{-5}	Carbamoyl-phosphate synthase large chain
BC3884	0.1	10^{-5}	Dihydroorotate dehydrogenase, catalytic subunit
BC3883	0.1	10^{-5}	Orotidine 5'-phosphate decarboxylase
BC4150	0.1	10^{-5}	Arginine-binding protein
BC3885	0.1	10^{-5}	Dihydroorotate dehydrogenase electron transfer subunit
BC4366	0.1	10^{-5}	Cystathionine beta-lyase
BC4915	0.2	10^{-5}	PaaI_thioesterase operon protein 2
BC2290	0.2	10^{-5}	Methylmalonate-semialdehyde dehydrogenase
BC1421	0.3	10^{-5}	Phosphoadenosine phosphosulfate reductase
