# Supplementary material for: Distinct Roles of ComK1 and ComK2 in Gene Regulation in Bacillus cereus
Source: PLoS One. 2011 Jul 1;6(7):e21859. doi: 10.1371/journal.pone.0021859 (PMC3128618; doi:10.1371/journal.pone.0021859)
Supplement: Table S3 — Summary of transcriptional changes in B. cereus ATCC15479 upon overexpression of comK2 . The top 20 genes significantly up- or down-regulated are shown in the table. The complete list of transcriptional changes is available at the Gene Expression Omnibus database under the accession number GSE27267. a The ratio of gene expression is shown. Ratio: expression in the comK2 overexpressed samples over control samples. b Bayesian p value. (PDF) [file pone.0021859.s004.pdf]

Table S3 Summary of transcriptional changes in *B. cereus* ATCC15479 upon overexpression of *comK2*. The top 20 genes significantly up- or down-regulated are shown in the table. The complete list of transcriptional changes is available at the Gene Expression Omnibus database under the accession number GSE27267. [a] The ratio of gene expression is shown. Ratio: expression in the *comK2* overexpressed samples over control samples. [b] Bayesian *p* value

Putative ComK2-upregulated genes.

Locus tag	*Expression ratio*[a]	*Significance p-value*[b]	*Annotation*[c]
**Upregulated**			
BC4930	163.9	10^{-9}	Transcriptional regulator, DeoR family
BC5250	139.1	10^{-9}	Competence transcription factor (comK2)
BC4929	82.7	10^{-9}	Macrolide-efflux protein
BC5247	82.2	10^{-7}	hypothetical protein
BC5251	64.9	10^{-7}	RNA polymerase sigma factor
BC5248	49.8	10^{-5}	hypothetical protein
BC0955	37.8	10^{-6}	Phage replication protein
BC0956	35.2	10^{-7}	Replicative DNA helicase
BC4931	22.1	10^{-7}	hypothetical Cytosolic Protein
BC4937	15.3	10^{-6}	hypothetical protein
BC0596	10.2	10^{-6}	Zinc-transporting ATPase
BC5027	9.4	10^{-5}	Protein erfK/srfK precursor
BC3399	9.1	10^{-5}	hypothetical protein
BC4935	8.3	10^{-5}	HESB protein
BC2271	7.9	10^{-5}	N-acetylmuramoyl-L-alanine amidase
BC3640	7.7	10^{-5}	Glyoxalase/Bleomycin resistance protein/Dioxygenase superfamily
BC0958	7.3	10^{-5}	Transcriptional regulator
BC5159	7.3	10^{-5}	Thioredoxin reductase
BC3690	7.3	10^{-5}	LexA repressor
BC0957	7.2	10^{-5}	hypothetical protein

Putative ComK2-downregulated genes.

Locus tag	*Expression ratio*[a]	*Significance p-value*[b]	*Annotation*[c]
**Downregulated**			
BC4127	0.06	10^{-7}	Acetylornithine aminotransferase
BC2289	0.07	10^{-7}	3-hydroxyisobutyrate dehydrogenase
BC4126	0.07	10^{-7}	Ornithine carbamoyltransferase
BC4131	0.08	10^{-6}	hypothetical protein
BC4128	0.08	10^{-7}	Acetylglutamate kinase
BC2290	0.1	10^{-7}	Methylmalonate-semialdehyde dehydrogenase (acylating)
BC4129	0.1	10^{-7}	Glutamate N-acetyltransferase
BC4630	0.1	10^{-6}	Argininosuccinate synthase
BC2288	0.1	10^{-6}	Acyl-CoA dehydrogenase
BC2252	0.1	10^{-6}	hypothetical Cytosolic Protein
BC2287	0.1	10^{-6}	Methylisocitrate lyase
BC2251	0.1	10^{-6}	Lysine 2,3-aminomutase
BC0745	0.1	10^{-6}	Hydroxymethylpyrimidine-binding protein
BC2284	0.1	10^{-6}	hypothetical Cytosolic Protein
BC0252	0.1	10^{-6}	4-hydroxyphenylpyruvate dioxygenase
BC2190	0.1	10^{-6}	Penicillin-binding protein
BC4150	0.1	10^{-6}	Arginine-binding protein
BC2551	0.1	10^{-6}	Transcriptional activator tenA
BC0253	0.1	10^{-6}	Fumarylacetoacetase
BC4148	0.1	10^{-6}	Arginine transport ATP-binding protein artP
